# Epigenetics of Estrogen Receptor Signaling: Role in Hormonal Cancer Progression and Therapy

**DOI:** 10.3390/cancers3021691

**Published:** 2011-03-29

**Authors:** Monica Mann, Valerie Cortez, Ratna K. Vadlamudi

**Affiliations:** 1 Department of Cellular and Structural Biology, UTHSCSA, 7703 Floyd Curl Drive, San Antonio, TX 78229, USA; E-Mails: mannm3@uthscsa.edu (M.M.); cortezv@uthscsa.edu (V.C.); 2 Department of Obstetrics and Gynecology, UTHSCSA, 7703 Floyd Curl Drive, San Antonio, TX 78229, USA

**Keywords:** estrogen receptor, histone modifications, epigenetics, breast cancer, hormonal cancers

## Abstract

Estrogen receptor (ERα) signaling plays a key role in hormonal cancer progression. ERα is a ligand-dependent transcription factor that modulates gene transcription via recruitment to the target gene chromatin. Emerging evidence suggests that ERα signaling has the potential to contribute to epigenetic changes. Estrogen stimulation is shown to induce several histone modifications at the ERα target gene promoters including acetylation, phosphorylation and methylation via dynamic interactions with histone modifying enzymes. Deregulation of enzymes involved in the ERα -mediated epigenetic pathway could play a vital role in ERα driven neoplastic processes. Unlike genetic alterations, epigenetic changes are reversible, and hence offer novel therapeutic opportunities to reverse ERα driven epigenetic changes. In this review, we summarize current knowledge on mechanisms by which ERα signaling potentiates epigenetic changes in cancer cells via histone modifications.

## 1. Introduction

The steroid hormone estradiol plays an important role in the initiation and progression of breast cancer. The biological effects of estrogen are mediated by its binding to the structurally and functionally distinct estrogen receptors (ERα and ERβ) [1]. ERα is implicated as a key transcriptional regulator in breast cancer biology [2,3]. ERα functions as a ligand-dependent transcription factor that modulates gene transcription via direct recruitment to target gene chromatin. The transcription functions of ERα are shown to be influenced by several coactivators, including SRC1, SRC2, AIB1, PELP1, CBP, p300, PCAF, CARM1, PRMT1 and corepressors such as NCoR, SMRT and MTA1 [4-6]. ERα transcriptional outcome is regulated by a dynamic interaction of histone modifying enzymes, which are frequently associated with coregulators [7,8]. Evidence suggests that multiprotein complexes containing ERα, coactivators and histone modifying enzymes assemble in response to hormone binding leading to activation of transcription [9]. Estrogen stimulation induces several histone modifications at the ERα target gene promoters including acetylation, phosphorylation and methylation. The **‘***histone-code hypothesis***’** proposes combinatorial and/or sequential post-translational modification of histones, that is written by specific enzymes (‘*writers*’) and removed by others (‘*erasers*’) can be read by nuclear factors (‘*readers*’) to promote a variety of cellular processes by regulating gene expression [10]. The histone code hypothesis suggests that post-translational modifications confer specific functions and the modified histones recruit specialized proteins that facilitate defined functions [11]. The mechanism by which ERα targets and coordinates activities of kinases/phosphatases and histone modifying enzymes is poorly understood. Estrogen-ERα signaling has been traditionally implicated in the stimulation of transcription. Several acetylases/deacetylases and methylases/demethylases interact with ERα directly or indirectly and facilitate the necessary histone modifications. ERα's ability to modulate epigenetic changes by regulating writers, erasers and readers of epigenetic modifications and the reversible nature of these modifications provides a unique therapeutic opportunity to design novel drugs/small molecular inhibitors for treating hormonal cancers. In this review, we summarized the key evidence that links ERα to the regulation of histone modifying enzymes and readers of histone modifications and discuss the possibility of targeting these pathways for therapeutics.

## 2. Mechanisms of ERα-mediated Histone Modifications

### 2.1. Acetylation/deacetylation

Acetylation and deacetylation of conserved lysine residues present in histone tails have been suggested as a mechanism by which ERα modifies chromatin structure [12]. Acetylated histones are usually associated with transcriptionally active chromatin, while deacetylated histones are associated with inactive chromatin [13]. ERα coactivators like SRC1, and AIB1 have been shown to possess histone acetyltransferase activity [14]. In addition, ERα associates with and modulates functions of general acetyltransferases including p300/CBP and p300/CBP-associated factor (PCAF) [2]. ERα -mediated deacetylation is accomplished by recruitment of histone deacetylases (HDACs), which are indirectly recruited to ERα target genes through multi-subunit corepressor complexes. The HDAC1/2-containing corepressor complex is the main route by which deacetylation of chromatin-associated histones takes place; the key adaptor protein in this complex is Sin3 [15]. ERα also utilizes corepressor complexes such as nuclear receptor corepressor (NCOR), silencing mediator of retinoid and thyroid hormone receptors (SMART) and the metastasis-associated 1 (MTA1) protein that associate with histone deacetylases [16]. Ligand-dependent corepressor (LCOR) is recruited to ERα target genes and interacts with ERα and HDAC6. Studies employing siRNA targeting HDAC6 and LCOR indicated that HDAC6 may function with LCoR on some ERα target genes (such as IGFBP4, ADORA1 and CYP26B1) as part of a feedback loop to regulate estrogen-dependent gene regulation in breast cancer cells [17]. A recent report showed that HDAC7 and FoxA1 interactions play a role in estrogen mediated repression of a subset of ERα target genes [18]. Collectively, these results suggest that ERα achieves histone acetylation modifications at target gene promoters using several coregulators.

### 2.2. Phosphorylation/dephosphorylation

In addition to its well established role in nuclear actions, ERα signaling also activates a number of kinases in the extranuclear compartment including protein kinase B (AKT) and extracellular signal-regulated protein kinase (ERK) [19]. Hormonal stimulation promotes alterations in the phosphorylation of specific residues in histone tails via modulation of these extranuclear kinases. Estrogen-ERα signaling activates mitogen-activated protein kinase (MAPK) cascades that transmit and amplify signals involved in cellular proliferation and apoptosis. Of the three major MAPK pathways in human tissues, the one involving ERK-1 and -2 is most relevant to breast cancer [20]. Estrogen-ERα signaling also activates Src-MAPK and Src-AKT pathways [21,22]. Since kinases that phosphorylate core histones (Msk1 and Msk2) and histone H1 (Cdk2) are downstream substrates of MAPK and AKT, ERα-mediated extranuclear signals can influence their downstream chromatin targets [23,24]. Estrogen-ERα signaling also regulates the expression and function of several phosphatases including PP1, PP2A and PDXP [25,26]. These results suggest that ERα-extranuclear signaling has the potential to modulate epigenetic modifications.

### 2.3. Methylation/demethylation

Histone methylation is crucial for regulating chromatin structure, gene transcription and the overall epigenetic state of the cell. The methylation of histones is a key regulatory signal in ERα-mediated gene expression and histone methylation-dependent mechanisms impose ligand dependency for gene activation [27]. Unlike acetylation, which is associated with gene activation, the consequence of histone methylation appears to be site dependent. For example, H3K4 methylation is linked with activation, while H3K9 methylation correlates with repression [28,29]. The recent discovery of lysine-specific demethylase 1 (KDM1, also designated as LSD1) suggests that histone methylation is reversible [30]. Recent studies showed that KDM1, which demethylates H3K4 and H3K9, is recruited to a significant fraction of ERα target genes and is required to demethylate proximal histones to enable ERα-mediated transcription [27]. Enhancer of Zeste homolog 2 (EZH2) is a histone methyltransferase and a polycomb group protein that catalyzes the histone modification of H3K27me3. Arginine methylation of histone tails occurs by the transfer of a methyl group to guanidine nitrogen of arginine catalyzed by the PRMT family of arginine methyltransferases. Type I PRMTs catalyze the formation of asymmetric dimethylarginine (me2a) while Type II PRMTs form symmetric dimethylarginine (me2s). Some of the modifications include the activation mark H3R2me2a by PRMT1, H3R17me2a and H3R26me2a by CARM1/PRMT4, as well as repressive marks H3R2me2a by PRMT6, H4R3me2s and H3R8me2s by PRMT5 [31,32]. H3R3me2a is antagonistic to the activation mark H3K4me3 which is catalyzed by the MLL1 complex. This is a very stable mark which at this point is not clear whether it can be completely enzymatically reversed [32]. Jumonji domain-containing protein (JMJD6) was believed to be a demethylase of asymmetric and symmetric H3R2me2 and H4R3me2 [33]. However, recently it was identified as a lysine hydroxylase without any detectable demethylase activity [34]. The methylarginine residue can also undergo a nonreversible conversion to citrulline through deamination by the peptidylarginine deiminases (PADIs) [35]. PADI4 is a novel transcriptional repressor that targets multiple sites including H3R17 and H4R3 catalyzed by CARM1 and PRMT1 respectively [36]. The activity of PADI4 is associated with the transcriptional regulation of estrogen responsive genes in MCF7 cells by recruitment to ERα promoters resulting in a decrease of ERα mediated gene induction [37]. PADI4 and HDAC work together to create a repressive chromatin state at the *TFF1* promoter [35]. Collectively, these studies suggest that ERα interacts with a variety of methylases and demethylases and deregulation of these enzymes may have implications on ERα target gene activation.

## 3. ER*α* Regulation of Histone Modifiers and Readers

### 3.1. ERα regulation of acetylases and deacetylases

ERα transcriptional outcome is shown to be regulated by a dynamic interaction of histone acetyltransferases and histone deacetylases, which are generally associated with coactivators and corepressors, respectively [7]. ERα signaling induces dramatic hyperacetylation at endogenous target genes through HAT activity utilizing p300/CBP to acetylate ERα coregulators [38]. ERα exerts a positive feedback role promoting induction of *hCYP19* gene transcription contributing to local estrogen synthesis by promoting increased acetylation in the hCYP promoter [39]. ERα also enhances the recruitment of MTA-1, a component of the histone deacetylase and nucleosome remodeling complex (NuRD), in a ligand and growth factor signaling dependent manner and such recruitment may involve an attenuation of ERα signaling [40]. Although estrogen-ERα signaling has been traditionally implicated in the stimulation of transcription, several recent studies using microarray and ChIP methodology indicated that more than half of the ERα transcriptome is repressed [41-43]. The mechanism by which ERα achieves differential regulation is elusive; however, it is suspected that differential cofactor recruitment and local chromatin modifications including deacetylation may play a role. EZH2 is a binding partner of REA (repressor of estrogen receptor activity) and this interaction is needed for its recruitment to specific target genes and repression of estrogen-dependent transcription. The inhibition of EZH2 by siRNA results in an increase of estrogen-dependent transcription [44]. The activity of PADI4 is associated with the transcriptional regulation of ERα responsive genes by being recruited to ERα promoters and modifying arginine residues on histones causing repression of ERα-mediated gene induction [37]. PADI4 and HDAC work together to create a repressive chromatin state at the *TFF1* promoter [35]. HDAC6, an estrogen target gene, expression levels correlate with better prognosis and response to endocrine therapy in breast cancer patients [45]. Class I and II HDACs can reverse p300-mediated acetylation in ERα, thereby inhibiting ERα-dependent gene transcription [46]. SIRT1, a class III HDAC, regulates ERα repression as well as ERα target gene expression [47].

### 3.2. ERα regulation of kinases and phosphatases

Using estrogen dendrimers and microarray analysis, it was demonstrated that around 25% of estrogen-regulated genes could be activated by ERα-extranuclear signaling pathways emphasizing the importance of these pathways in the activation of ERα target genes [48]. Cell cycle-dependent phosphorylation of histone H3 in both ovarian granulosa and breast cancer cells is driven by estrogen, acting through the oncogenic kinase, Aurora B [49]. Membrane-associated ERα signaling regulates EZH2 via phosphorylation at S21 by constitutively activated AKT resulting in a decrease of H3K27me3 in hormone-responsive cells [50]. Estrogen is implicated in the overexpression of Aurora A/B and the deregulation of Aurora kinase protein substrates is involved in eliciting the alterations observed during oncogenesis [51]. ERα activates ERK2, resulting in colocalization at chromatin binding sites across the genome of breast cancer cells and enables ERK2 modulation of estrogen-dependent gene expression and proliferation programs. This study revealed a novel paradigm with convergence of ERK2 and ERα at the chromatin level that positions this kinase to support nuclear receptor activities in a direct manner [52].

### 3.3. ERα regulation of methylases and demethylases

Mixed lineage leukemia histone methylases (MLL1-4) are H3K4 methyltransferases [53] and H3K4 trimethylation correlates with ERα transcriptional activation [54]. ERα also recruits MLLs to the HOXC13 promoter and the knockdown of MLLs suppresses the estrogen-induced activation of HOXC13 [55]. Another study showed direct interaction of ERα with MLL2 plays a central role in the growth of ERα positive cells [56]. SMYD3 directly interacts with the ligand binding domain of ERα and is recruited to its target gene promoters *TFF1* and *GREB1C* upon gene induction, catalyzing H3K4me3 [57]. Optimal ERα transcription requires removal of methyl marks such as H3K9 facilitated by demethylase KDM1 and the addition of methyl marks such as H3K4me2 [58]. KDM1 is widely recruited to active promoters in estrogen-stimulated cells and opposes the silencing function of H3K9 methyltransferase SETDB1 [59]. Hormonal stimulation also induce the cyclic recruitment of CARM1 and PRMT1 to the ERα target genes and both are coactivators of ERα [60]. PADI4 is recruited to the *TFF1* promoter before the loss of H3R17me2a and deamination of methylarginines at R2, R8, R17 and R26 [35,37]. CARM1 is essential for estrogen-induced cell cycle progression in MCF7 cells and the cell cycle transcriptional regulator E2F1. However, H3R17me2 of E2F1 by CARM1 is dependent on the oncogenic coactivator AIB1 [61]. CARM1 is recruited to the *TFF1* promoter during transcriptional activation and deamination by PADI4 inhibits methylation by CARM1.

### 3.4. ERα regulation of readers of histone modifications

A recent study using a protein domain microarray approach identified the Tudor domain-containing protein (TDRD3) as a reader of histone arginine methyl marks. Importantly, hormonal treatment induces TDRD3 recruitment to ERα target genes, and enhances ERα transcriptional activation. These results suggest that TDRD3 serves as an effector molecule that promotes ERα transcription by binding methylarginine marks on histone tails [62]. Menin, a component of the MLL complex, is a transcriptional coactivator of ERα serving as a link between MLL recruitment and ERα-mediated transcription. Menin is recruited by the activation of *TFF1* resulting in increased H3K4 methylation [63]. Recent study results indicate that ERα-coregulator proline glutamic acid leucine rich protein-1 (PELP1) is a novel KDM1-interacting protein. PELP1 functions as a reader of dimethyl histone modifications. Hormonal stimulation enhances PELP1 interactions with KDM1 and PELP1-KDM1 interactions play an essential role in histone methyl modifications at ERα target genes [64]. Additionally, a recent study identified PELP1 as a component of the MLL1 methyltransferase complex [65].

## 4. Deregulation of Histone Modifying Enzymes in Hormonal Cancers

The human ERα is implicated in hormonal cancer initiation and progression [2,8,66]. Current endocrine therapy for ERα-positive hormonal cancer involves modulating the ERα-pathway using either antiestrogens (AEs) or aromatase inhibitors (AIs). Despite the positive effects, *de novo* and/or acquired resistance to endocrine therapies frequently occurs. While mechanisms for hormonal therapy resistance remain elusive, emerging data suggest that resistance can be caused by acquisition of epigenetic modifications on ERα and its target gene promoters [67-69]. Some evidence also implicates activation of MAPK- and AKT-signaling pathways in activating ERα and its downstream pathways in the absence of estrogen and thus these pathways represent new targets for drug therapy [70,71]. Estrogen stimulation promotes histone modifications at ERα target gene promoters [72]. SMYD3, the H3K4me methyltransferase, is overexpressed in breast cancer causing dysregulation of the WNT signaling pathway [73]. The MLL complex also plays a role in breast cancer. The oncogenic transformation caused by this complex can be blocked by knockdown of the MLL component PRMT1 [74].

EZH2 overexpression in breast cancer is associated with larger tumors, higher histological grade and reduced overall patient survival [50,75]. High expression of EZH2 is sufficient to induce oncogenic potential in a xenograft model [76]. There is a strong correlation between EZH2 expression levels and increased tumor cell proliferation [77]. EZH2 is essential for promoting chemotherapy resistance in cancer cells *in vitro* and *in vivo*, and EZH2 could be a potential novel epigenetic target to overcome drug resistance [78]. Also, the frequent overexpression of EZH2 in human epithelial ovarian cancer cells promotes cellular proliferation and invasive ability, further supporting its possibility as a novel therapeutic target [79]. CARM1 is an essential part of the estrogen-stimulated breast cancer growth downstream of ERα and its aberrant expression as well as PRMT1 overexpression is linked to breast cancer [61,80]. PADI4 is overexpressed in malignant tumors but not in benign tumors [37]. ERα is critical for demethylase JMJD2B induction in hypoxia which is critical for breast cancer cell survival, is highly expressed in ERα-positive primary breast cancers and is an adverse prognostic factor in hypoxic breast cancers [81].

Deregulation of AIB1, an ERα coregulator with intrinsic acetylase activity, was reported in breast tumors [82,83]. Elevated amounts of coregulators SRC-2 and CBP have been reported in intraductal carcinomas compared to normal mammary tissue [84]. ERα coregulatory proteins such as SRC1 with histone modifying activity have also been suggested to play a role in the generally observed tissue-specific effects of tamoxifen [85]. AIB1 knockout mice studies demonstrated that normal expression of coactivator AIB1 is required for the initiation of tumorigenesis by carcinogens and oncogenes [86,87]. Overexpression of AIB1 in the mouse mammary gland promotes tumorigenesis suggesting that ERα coregulators have oncogenic potential [88]. Similarly, in mammary epithelium dysregulation of another ERα coregulator MTA1 that associates with deacetylase complexes caused increased cell proliferation, hyper-branched ductal structure formation and precocious development. It also resulted in the development of hyperplastic nodules and mammary gland tumors in virgin mice [89]. PELP1, an ERα coregulator and reader of epigenetic modifications plays an important role in ERα signaling [90]. PELP1 is a recently discovered proto-oncogene [91] that exhibits aberrant expression in many hormonal cancers [90] and is a prognostic indicator of decreased survival in breast cancer and disease-free intervals when over-expressed [92].

## 5. Therapeutic Potential of Targeting Histone Modifying Enzymes for Hormonal Driven Cancers

Epigenetic modifications are reversible, and drugs targeting epigenetic modifiers which are often overexpressed in hormonal cancers, can be potentially used for therapeutic targeting. Inhibition of SIRT1 deacetylase activity by either pharmacological inhibitors or genetic depletion impairs ERα-mediated signaling pathways [47]. Research performed over the last decade has highlighted the role of HDAC inhibitors as modulators of transcriptional activity. A new class of therapeutic agents in ERα-driven as well as ERα-therapy resistant cells [93] has led to the initiation of several clinical trials combining HDAC inhibitors with hormonal therapy [94,95]. Clinical trials show that HDAC inhibitors have varying antitumor activity, the FDA-approved HDAC inhibitors (SAHA/vorinostat and depsipeptide) had significant clinical benefits [96]. SAHA promotes ERα degradation by a proteasome-mediated mechanism implicating SAHA as a suitable pharmacological agent for the depletion of ERα in breast tumors [97]. Currently SAHA is given in combination with tamoxifen for patients with advanced breast cancer for whom anti-hormonal therapy has been ineffective. Recent studies also showed that HDAC inhibitors (LBH589/panobinostat) function as potent inhibitors of aromatase expression. Results from this study demonstrated synergistic interaction between LBH589 and letrozole to suppress proliferation of hormone-responsive breast cancer cells [98]. Entinostat (ENT), another HDAC inhibitor is shown to trigger re-expression of ERα and aromatase in breast cancer cells. Preclinical studies showed that ENT treatment restores letrozole responsiveness of ERα-negative tumors providing a strong rationale for clinical evaluation of combinatorial therapy of ENT and letrozole to treat ERα-negative and endocrine-resistant breast cancers [99].

Recent studies also showed combination therapy involving HDAC inhibitors with DNA methyltransferase-1 (DNMT1) inhibition is synergistically effective in inducing apoptosis, differentiation and/or cell growth arrest in many cancers including breast cancer [100]. DNMT1 inhibitor 5-aza-2′-deoxycytidine (AZA) and HDACi trichostatin A (TSA) induces ERα expression in ERα-negative breast cancer cells. Preclinical studies indicate AZA and TSA could restore sensitivity of the ERα-negative breast cancer cells to endocrine therapy *in vitro* and *in vivo* [101]. Zebularine, another DNMT inhibitor, inhibits the growth of breast cancer cells. Combination therapy with HDAC inhibitors (decitabine or vorinostat) significantly inhibited cellular proliferation and colony formation in breast cancer cells compared to either drug alone [102].

Preclinical studies also suggest that drugs targeting histone methylation would have therapeutic benefits in treating hormonal cancers. Dysregulation of arginine methylation or enzymes responsible for these modifications could be key events in estrogen-dependent cancers and thus these enzymes represent novel therapeutic targets [4]. Small molecule regulator of protein arginine methyltransferases (AMI-1) is shown to modulate nuclear receptor-regulated transcription from estrogen and androgen response elements, thus operating as a brake on certain hormone actions [103]. Recent studies demonstrated that F- and Cl-amidine, two potent PADI4 inhibitors, display micromolar cytotoxic effects towards several cancerous cell lines (HL-60, MCF7 and HT-29) with no effect on noncancerous lines implicating PADI4 inhibition as a novel epigenetic approach for the treatment of hormonal cancer [37]. A recent study showed that targeting EZH2 via siRNA could be used to reduce angiogenesis and ovarian cancer growth proving the feasibility for using EZH2 as an important therapeutic target [104]. Histone methyltransferases such as G9a are required to perpetuate the malignant phenotype [105] and G9a inhibitor BIX-01294 (diazepin-quinazolin-amine derivative) may have therapeutic utility in breast cancer cells overexpressing the methyltransferases [106,107].

Histone methylation is shown to play a key role in ERα-mediated transactivation of target genes. Recent studies showed the histone demethylase KDM1 and ERα coregulator PELP1 play a role in regulating histone methyl marks at ERα target genes [64]. PELP1 deregulation alters histone methylation at ERα target genes, contributing to hormone-driven tumor progression [64] and therapy resistance [108]. In a recent study, we examined the therapeutic efficacy of treating breast tumor cells with Pargyline, an FDA approved drug to block KDM1 functions. The results from this study suggested that histone methyl modifications play a role in therapy resistance and targeting the KDM1 axis with Pargyline in combination with current endocrine therapies will improve therapeutic efficacy [109]. Polycomb-repressive complex 2 (PRC2)-mediated histone methylation plays an important role in aberrant cancer gene silencing. Preclinical studies using S-adenosyl homocysteine hydrolase inhibitor 3-Deazaneplanocin A (DZNep) induces efficient apoptotic cell death in cancer cells but not in normal cells. Mechanistic studies showed that DZNep effectively depletes cellular levels of PRC2 components EZH2, SUZ12 and EED and inhibits histone H3K27 methylation suggesting a unique feature of DZNep as a novel chromatin remodeling compound that could be used as a novel cancer therapeutic [110].

## 6. Conclusions and Future Directions

**Figure 1.**
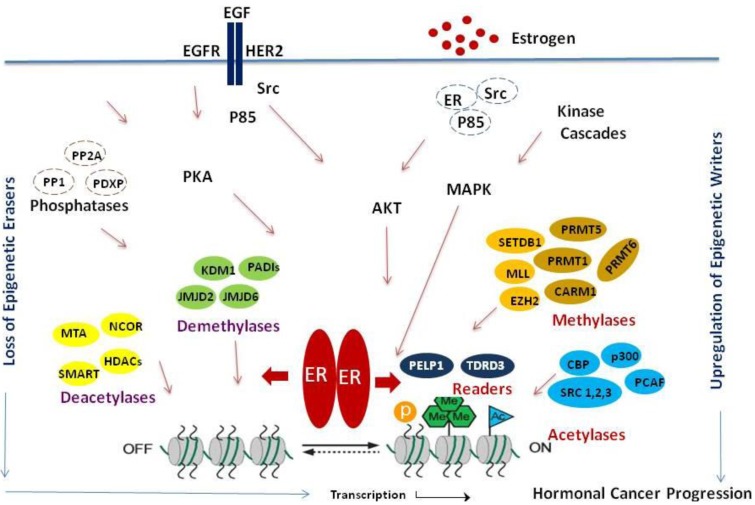
Schematic representation of the current understanding of ERα regulation of epigenetic modifications of histones. ERα–mediated transcription involves coordinated interactions of ERα with acetylases and deacetylases, methylases and demethylases. Ligand stimulation uniquely modulates ERα interactions with histone modifying enzymes. In addition, estrogen signaling has the potential to activate extranuclear signaling that activates several kinase cascades that either directly modifies histone tails or indirectly influence functions and/or recruitment of histone modifying enzymes. Deregulation of ERα-mediated epigenetic signaling will have implications in hormonal tumor progression and therapy resistance.

The emerging evidence strongly implicates the importance of ERα-mediated epigenetic modifications to ERα. Histone tail modifications play a significant role in ERα–mediated physiological functions as well as in cancer progression (Figure 1). In this review, we focused on summarizing recent findings that relate to ERα-mediated histone tail modifications, enzymes that mediate ERα-mediated epigenetic modifications on histone tails, deregulation of these enzymes in hormonal cancers and its implications in ERα signaling. Because of the evolving evidence connecting histone modifications and hormonal cancer progression/therapy resistance, the epigenetic enzymes that play a role in ERα signaling represent promising targets for hormonal cancer treatment. Further, combinatorial treatment of HDACi and/or histone methylase inhibitors along with currently used hormonal therapy regimen(s) may be a useful tool in treating therapy resistant cancers. However, future improvements in therapeutic targeting of ERα epigenetic modifications will depend upon a better understanding of: (1) molecular basis by which epigenetic modifications play a role in cancer progression; (2) epigenetic code during progression and therapy resistance using the emerging Chip-Seq methodologies; (3) prognostic significance of the epigenetic marks in hormonal cancer progression and (4) development of specific molecular inhibitors targeting epigenetic modifiers.
